# The Impact of Human Service Provider Quality on the Personal Outcomes of People With Intellectual and Developmental Disabilities

**DOI:** 10.3389/fresc.2021.780168

**Published:** 2022-01-17

**Authors:** Carli Friedman

**Affiliations:** The Council on Quality and Leadership (CQL), Towson, MD, United States

**Keywords:** people with intellectual and developmental disabilities, personal outcomes, quality of life, quality improvement, human service providers

## Abstract

**Background:**

Quality of life is multidimensional—influenced by individual, organizational, and environmental factors. As such, when examining personal outcomes, it is also important to consider meso and macro factors that contribute to people with intellectual and developmental disabilities' (IDD's) quality of life. While it is widely acknowledged that organizational factors contribute to people's quality of life, there is less research directly examining how the *quality* of human service providers contributes to people with IDD's personal outcomes. For these reasons, the aim of this study was to explore the relationship between provider quality and people with IDD's personal quality of life outcomes.

**Methods:**

Using a multilevel linear regression we analyzed secondary Personal Outcome Measures® (personal outcomes) and Basic Assurances® (provider quality) data from 2,900 people with IDD served by 331 human service providers.

**Results:**

People with IDD's personal outcomes, regardless of their support needs or other demographics, were significantly impacted by the quality of the human service providers they received services from—the higher the quality of the provider, the more personal outcomes they had present. In addition, the following demographic covariates were correlated with personal outcomes: gender; race; complex support needs; residence type; and organizations that offered therapy services.

**Discussion:**

While quality improvement initiatives may require a great deal of cost and time commitment from providers, our findings suggest the effort translates to improved personal outcomes among people with IDD. The ultimate goal of service providers should be improvement of quality of life among those they support.

## Background

Quality of life is based on “common human experiences and unique, individual life experiences” [(1), p. 462] while also giving “sense of reference and guidance from the individual's perspective, focusing on the person and the individual's environment” [(2), p. 2]. Disability quality of life measures were originally developed to examine the “burden” of disabilities (3); however, in recognition that the person, family, community, and society all impact quality of life, disability quality of life measures have since broadened to examine physical, material, and emotional well-being, relationships, personal development, rights, inclusion, and self-determination (4). As such, in contrast to process measures that often focus on compliance and regulations, disability quality of life measures should focus on an individualized person-centered definition of quality of life, also called personal outcomes (5). In fact, the Centers for Medicare and Medicaid Services (CMS) reinforced the importance of personal outcomes with the implementation of the Medicaid HCBS settings rule (CMS 2249-F/2296-F); CMS (6) explained, the HCBS Settings Rule would “establish a more outcome-oriented definition of home and community-based settings, rather than one based solely on a setting's location, geography, or physical characteristics” (p. 2).

Quality of life is multidimensional—influenced by individual, organizational, and environmental factors (7–10). Therefore, it is important when examining personal outcomes to also consider meso and macro factors that contribute to people with intellectual and developmental disabilities' (IDD's) quality of life. In fact, Simões and Santos (7) note, “it can be said that quality of life may have less to do with a presence of an ID [intellectual disability] and more to do with the opportunities that improve individual's participation in community-based settings. Thus, the supports have a crucial influence on individual's quality of life” (p. 391).

Organizational characteristics and factors, related to the services people with IDD receive and their human service provider/s, contribute to people with IDD's quality of life (10, 11). Examples of organizational factors that can impact people with IDD's personal outcomes include: staff qualifications, satisfaction, leadership, and turnover; residence types and sizes; day activities; organizational culture; person-centered practices; organization size; and locations of service delivery (7, 8, 11, 12). For example, Claes and Van Hove (10) found when staff involved, included, and empowered people with ID, their personal outcomes improved. Moreover, Gómez et al. (8) found differences in personal outcomes among people with disabilities based on not only their individual characteristics, but also the types of services they received. In addition, Flynn et al.'s (11) meta-analysis revealed Active Support—staff training about engagement, independence, and self-determination—lead to increases in the overall engagement of people with ID.

In fact, quality IDD services can be defined by the degree to which human service organizations promote and maximize personal outcomes (5, 13, 14). While quality IDD services used to be defined in relation to compliance, regulatory standards, and organizational processes, there has since been a shift to recognizing quality as “responsiveness to people's outcomes… [and] the continuous discovery and fulfillment of [people with IDD's] needs and desires” [(13), p. 295–300]. Moreover, the United States Department of Health and Human Services notes, “Quality is directly linked to an organization's service delivery approach or underlying systems of care… resources (*inputs*) and activities carried out (*processes*) are addressed together to ensure or improve quality of care (*outputs/outcomes*)” [emphasis original; (14), p. 1].

While it is widely acknowledged that organizational factors contribute to people with IDD's quality of life (4, 7–9, 13), to our knowledge, there is little research *directly* examining how the quality of human service providers contributes to people with IDD's quality of life—their personal outcomes. For these reasons, the aim of this study was to explore the relationship between human service provider quality and personal quality of life outcomes of people with IDD. To do so, we analyzed data from 2,900 people with IDD served by 331 human service providers.

## Materials and Methods

### Data and Participants

This was a secondary data analysis. All data were originally collected from between January 2015 to August 2021 from organizations that provide services to people with IDD, including: residential services; employment and other work/day services; family and individual supports; behavioral health care; service coordination; case management; non-traditional supports (micro-boards and co-ops); and human services systems. The data included 2,900 people with IDD served by 331 human service providers.

Two levels of data were included in the study: level 1 was individual people with IDD, while level 2 was human service providers. In terms of level 1, the average age of people with IDD was 44.79 (*SD* = 16.26; Table 1). Slightly more than half of the people with IDD were men (54.7%). Most participants were White (75.7%) and communicated primarily through verbal/spoken language (84.7%). The most common form of decision-making authority (guardianship) was full/plenary guardianship (41.9%), with fewer people having independent decision-making (32.4%), assisted decision-making (22.9%), or other forms of decision-making (2.7%). In terms of complex support needs, 9.3% of people with IDD had complex medical support needs (12+ h of skilled nursing care), 18.2% comprehensive behavior support needs (24-h supervision due to risk of harm), and 7.0% *both* support needs. About half of participants (47.5%) lived in provider owned- or operated-homes; the next most common settings were their own home (22.5%), and family homes (16.6%).

**Table 1:** Demographics.

**Characteristics**	***n***	***%***
**Individuals (level 1;** ***n*** **=** **2,900)**		
Age [*n* = 2,369; *M* (*SD*)]	44.79 (16.26)	
Gender (*n* = 2,870)		
Man	1,570	54.7%
Woman	1,300	45.3%
Primary communication method (*n* = 2,870)		
Verbal/spoken language	2,438	84.7%
Other	441	15.3%
Decision-making authority (*n* = 2,851)		
Independent decision-making	925	32.4%
Assisted decision-making	653	22.9%
Full/plenary guardianship	1,195	41.9%
Other	78	2.7%
Race (*n* = 2,864)		
White	2,168	75.7%
Indigenous	82	2.9%
Asian	15	0.5%
Black	507	17.7%
Latinx	60	2.1%
Other	12	0.4%
Multiracial	20	0.7%
Complex support needs (*n* = 2,509)		
None	1,643	65.5%
Complex medical support needs	234	9.3%
Comprehensive behavior support needs	456	18.2%
Both	176	7.0%
Residence (*n* = 2,848)		
Provider owned/operated home	1,352	47.5%
Own home	642	22.5%
Family's home	473	16.6%
Host home or family foster care	102	3.6%
State HCBS group home	73	2.6%
State ICF/DD	27	0.9%
Private ICF/DD	70	2.5%
Nursing home	17	0.6%
Other	92	3.2%
**Providers (level 2;** ***n*** **=** **331)**		
Unduplicated number of people supported [*n* = 299; *M* (*SD*)]	796.52 (1,163.32)	
Geographic region (*n* = 299)		
Urban only	42	14.0%
Rural only	102	34.1%
Both urban and rural	155	51.8%
Services provided		
Behavior support services (*n* = 299)	136	45.5%
Therapies (e.g., psychology, physical therapy, occupational therapy, speech/language; *n* = 299)	113	37.8%
Staffed residential supports (*n* = 299)	228	76.3%
Host home, family foster care, or companion home (*n* = 299)	81	27.1%
In-home supports (own home or family home; *n* = 299)	199	66.6%
Community-based employment (*n* = 299)	256	85.6%
Community-based day activities (*n* = 299)	208	69.6%
In-home day activities (*n* = 299)	132	44.1%
Facility-based work/day activities (*n* = 299)	161	53.8%
Respite care (*n* = 299)	149	49.8%
Recreational activities (*n* = 299)	110	36.8%
Transportation activities (*n* = 299)	172	57.5%
Independent support coordination (*n* = 299)	52	17.4%

In terms of level 2 demographics, the 331 providers supported an average of 796.52 unduplicated people (*SD* = 1,163.32). About half (51.8%) provided services in *both* urban and rural areas, 34.1% in only rural areas, and 14.0% only urban areas. The most common types of services they provided were: community-based employment (85.6%); staffed residential supports (76.3%); community-based day activities (69.6%); and, in-home supports (66.6%). All of the individual (level 1) and provider (level 2) demographic variables served as covariates in the analyses.

### Measures and Variables

#### Personal Outcomes: Personal Outcome Measures® (Level 1: Individual)

Data about people with IDD's quality of life—their personal outcomes—came from the Personal Outcome Measures® (15). The Personal Outcome Measures® is a validated, person-centered quality of life tool (16). The Personal Outcome Measures® was developed in 1993 based on focus groups with people with disabilities, family members, and other key stakeholders about what really mattered in people with disabilities' lives. The tool has since been refined through pilot testing, commission of research and content experts, a Delphi survey, feedback from advisory groups, validity and reliability testing, and 30 years of administration (13, 15, 16). For example, the most recent validity testing used a principal component analysis to indicate construct validity and internal consistency (16). In addition, interviewers are required to pass interrater reliability tests with expert interviewers with scores of 85% or higher before being certified to conduct interviews.

The most recent version of the Personal Outcome Measures® (2017) includes 21 indicators (areas of quality of life; see Table 2) organized into five factors: My Human Security; My Community; My Relationships; My Choices; and, My Goals. Personal Outcome Measures® administration occurs in three stages. During the first stage, a certified reliable interviewer has an in-depth conversation with the person with IDD about each of the indicators, following specific open-ended prompts. Next, the interviewer speaks with someone who knows the person with IDD well and knows about their organizational supports, and asks them questions about individualized supports and outcomes to fill in any gaps. In the third and final stage, the interviewer may participate in observations or conduct record reviews if needed; otherwise, they complete decision trees [see The Council on Quality and Leadership (15) for decision-trees] based on all information gathered to determine if outcomes are present (1) or not (0). The 21 different indicators are then summed to represent the total number of personal outcomes present for each person with IDD.

**Table 2:** Quality indicators.

**Personal Outcome Measures^®^ Indicators**	**Basic Assurances^®^ Indicators**
**My human security**	**Rights protection and promotion**
People are safe	The organization implements policies and procedures that promote people's rights
People are free from abuse and neglect	The organization supports people to exercise their rights and responsibilities
People have the best possible health	Staff recognize and honor people's rights
People experience continuity and security	The organization upholds due process requirements
People exercise rights	Decision-making supports are provided to people as needed
People are treated fairly	**Dignity and respect**
People are respected	People are treated as people first
**My community**	The organization respects people's concerns and responds accordingly
People use their environments	People have privacy
People live in integrated environments	Supports and services enhance dignity and respect
People interact with other members of the community	People have meaningful work and activity choices
People participate in the life of the community	**Natural support networks**
**My relationship** *s*	Policies and practices facilitate continuity of natural support systems
People are connected to natural supports	The organization recognizes emerging support networks
People have friends	Communication occurs among people, their support staff and their families
People have intimate relationships	The organization facilitates each person's desire for natural supports
People decide when to share personal information	**Protection from abuse, neglect, mistreatment and exploitation**
People perform different social roles	The organization implements policies and procedures that define, prohibit and prevent abuse, neglect
**My choices**	mistreatment and exploitation
People choose where and with whom to live	People are free from abuse, neglect, mistreatment and exploitation
People choose where to work People choose services	The organization implements systems for reviewing and analyzing trends, potential risks and sentinel events including allegations of abuse, neglect, mistreatment and exploitation, and injuries of unknown origin and deaths
**My goals**	Support staff know how to prevent, detect and report allegations of abuse, neglect, mistreatment and exploitation
People choose personal goals People realize personal goals	The organization ensures objective, prompt and thorough investigations of each allegation of abuse, neglect, mistreatment and exploitation, and of each injury, particularly injuries of unknown origin
	The organization ensures thorough, appropriate and prompt responses to substantiated cases of abuse, neglect, mistreatment and exploitation, and to other associated issues identified in the investigation
	**Best possible health**
	People have supports to manage their own health care
	People access quality health care
	Data and documentation support evaluation of health care objectives and promote continuity of services and supports
	Acute health needs are addressed in a timely manner
	People receive medications and treatments safely and effectively
	Staff immediately recognize and respond to medical emergencies
	**Safe environments**
	The organization provides individualized safety supports
	The physical environment promotes people's health, safety and independence
	The organization has individualized emergency plans
	Routine inspections ensure that environments are sanitary and hazard free
	**Staff resources and supports**
	The organization implements a system for staff recruitment and retention
	The organization implements an ongoing staff development program
	The support needs of individuals shape the hiring, training and assignment of all staff
	The organization implements systems that promote continuity and consistency of direct support professionals
	The organization treats its employees with dignity, respect and fairness
	**Positive services and supports**
	People's individual plans lead to person-centered and person-directed services and supports
	The organization provides continuous and consistent services and supports for each person
	The organization provides positive behavioral supports to people
	The organization treats people with psychoactive medications for mental health needs consistent with national standards of care
	People are free from unnecessary, intrusive interventions
	**Continuity and personal security**
	The organization's mission, vision and values promote attainment of personal outcomes
	The organization implements sound fiscal practices
	Business, administrative and support functions promote personal outcomes
	The cumulative record of personal information promotes continuity of services
	**Basic assurances system**
	The organization monitors Basic Assurances
	A comprehensive plan describes the methods and procedures for monitoring Basic Assurances

*Basic Assurances^®^ indicators are measured both in terms of systems and practices, resulting in 92 total datapoints*.

#### Provider Quality: Basic Assurances® (Level 2: Organizational)

Data regarding the quality of human service providers came from the Basic Assurances® (17). The Basic Assurances® is an organizational assessment of non-negotiable requirements for service and support providers, including health, safety, and human security metrics; the “*Basic Assurances*® looks at the provision of safeguards from the person's perspective. While the *Basic Assurances*® contain requirements for certain systems and policies and procedures, the effectiveness of the system or the policy is determined in practice, person by person” [(17), p. 8].

The Basic Assurances® was developed in 1971 (originally called “Standards for Services”) based on feedback from practitioners, providers, government personnel, advocacy organizations, people with disabilities, and parents about high quality service standards. Since then, it has undergone numerous revisions based on reviews by experts, pilot testing, a Delphi survey, development of a conceptual framework, stakeholder interviews, and 50 years of administration (17–20). To promote reliability, reviewers are required to pass interrater reliability tests with expert reviewers with scores of 85% or higher.

The most recent version of the Basic Assurances® (2015) contain 10 factors: Rights Protection and Promotion; Dignity and Respect; Natural Support Networks; Protection from Abuse, Neglect, Mistreatment and Exploitation; Best Possible Health; Safe Environments; Staff Resources and Supports; Positive Services and Supports; Continuity and Personal Security; and, Basic Assurances® System (a quality assurances monitoring system). Within the 10 factors are 46 different sub-topics, called indicators. For each of the 46 indicators (Table 2), both the *system*—“organizational supports that provide the structure for organizational practice” (e.g., policies and procedures)—and actual *practice*—“what is observed in daily operations… how an organization's supports are put into action” (i.e., implementation)—are examined and measured [(17), p. 9]; as a result, the total possible number of indicators present for a provider is 92.

To determine if systems and practices are present for each indicator, expert reviewers collect a number of data points. Sources of data include: interviews with organizational leadership; interviews with people with IDD; focus groups with people with IDD; focus groups with direct support professionals; reviews of the providers' data and records; reviews of organizational policies and regulations; and, observations of a variety of the provider's settings. Using all of these data, the expert reviewers, often working in teams of 2–4 for interrater reliability, determine if each of the indicators are present (1) or not (0) for each system and each practice [see The Council on Quality and Leadership (17) for probes for each indicator]. The 92 different indicator items are then summed to represent the total provider quality for each provider.

### Analyses

We first analyzed descriptive statistics (missing data were excluded from all analyses.). Then, to examine the impact of provider quality on the personal outcomes of people with IDD, we used a multilevel linear regression (linear mixed model; all assumptions were met). This method was used to account for the nested structure of the data between individuals with IDD (level 1; *n* = 2,900) and providers (level 2; *n* = 331). We first ran an intercept-only unconditional (null) model with only the total personal outcomes from the Personal Outcome Measures® serving as the primary outcome and the random intercept to examine variation in personal outcomes by providers; maximum likelihood estimation was used. In the second model, we entered all demographic variables—the covariate individual-level and provider-level demographic variables were added as fixed-effects. In the third and final model, provider quality from the Basic Assurances® was also added as a fixed-effect variable. Intraclass correlation coefficients (ICCs) were calculated for each model to indicate variance in personal outcomes attributed to different providers; ICC were calculated by dividing the intercept variance by the sum of the intercept and residual variance. Cohen's *f* ^2^ was calculated (21) for effect size for the final model.

## Results

### Descriptive Statistics

The people with IDD in the study had an average of 9.85 out of 21 possible personal outcomes present (*SD* = 5.09). Of people with IDD, 1.5% had 0 outcomes present, 18.8% between 1 and 5 outcomes, 34.8% between 6 and 10 outcomes, 27.9% between 11 and 15 outcomes, 13.9% between 16 and 20 outcomes, and 3.1% all 21 outcomes. Providers in the study had an average of 69.71 out of 92 possible total Basic Assurances® indicators present (*SD* = 11.66). Of the providers, 3.5% had between 30 and 42 indicators present, 10.1% between 43 and 55 indicators, 31.6% between 56 and 68 indicators, 39.7% between 69 and 81 indicators, and 15.1% between 82 and 92 indicators.

### The Relationship Between Provider Quality and People's Personal Outcomes

To explore if personal outcomes differed depending on provider quality, linear multilevel models were utilized. In the first unconditional (null) model, which was calculated without any covariates, the ICC indicated 35.0% of the total variation in personal outcomes is attributed to differences between providers (Table 3).

**Table 3:** The impact of organizational quality on personal outcomes: multilevel linear regression models.

**Predictors**	**Model 1: null model**	**Model 2: demographic model** **[B (95% CI)]**	**Model 3: provider quality** **[B (95% CI)]**
**Fixed effects**			
Intercept		11.07 (9.21–12.94)^***^	5.93 (2.96–8.90)^***^
Individual (level 1)			
Age		−0.001 (−0.01 to 0.01)	−0.006 (−0.02 to 0.007)
Woman (ref: man)		−0.66 (−1.03 to −0.28)^***^	−0.61 (−1.02 to −0.20)^**^
Primary communication method: other (ref: verbal/spoken language)		−0.18 (−0.72 to 0.36)	−0.16 (−0.76 to 0.45)
Decision-making authority (ref independent decision-making)			
Assisted decision-making		−0.71 (−1.20 to −0.23)^**^	−0.90 (−1.43 to −0.36)^**^
Full/plenary guardianship		−0.15 (−0.67 to 0.38)	−0.15 (−0.73 to 0.42)
Other		−0.51 (−1.63 to 0.61)	−0.54 (−1.77 to 0.68)
Race (ref: white)		
Indigenous		−1.42 (−2.59 to −0.24)^*^	−1.16 (−2.55 to 0.24)
Asian		−2.90 (−5.52 to −0.28)^*^	−1.40 (−4.32 to 1.53)
Black		−0.50 (−1.04 to 0.03)	−0.45 (−1.02 to 0.11)
Latinx		−0.07 (−1.57 to 1.44)	−0.16 (−1.73 to 1.40)
Other		−1.11 (−4.08 to 1.85)	−2.04 (−5.28 to 1.20)
Multiracial		−2.44 (−4.55 to −0.34)^*^	−2.37 (−4.64 to −0.09)^*^
Complex support needs (ref: none)			
Complex medical support needs		−0.36 (−1.02 to 0.30)	−0.31 (−1.03 to 0.41)
Comprehensive behavior support Needs		−0.99 (−1.52 to −0.46)^***^	−1.01 (−1.58 to −0.44)^***^
Both		−0.82 (−1.60 to −0.03)^*^	−0.83 (−1.66 to −0.01)^*^
Residence (ref: provider owned/operated home)			
Own home		1.90 (1.36–2.45)^***^	1.60 (0.98–2.22)^***^
Family's home		1.50 (0.88–2.11)^***^	1.44 (0.78–2.10)^***^
Host home or family foster care		2.51 (1.46–3.55)^***^	2.48 (1.32–3.63)^***^
State HCBS group home		−0.97 (−3.19 to 1.25)	−0.87 (−3.08 to 1.35)
State ICF/DD		0.28 (−1.15 to 1.70)	0.004 (−1.48 to 1.49)
Private ICF/DD		−2.49 (−5.26 to 0.27)	−1.73 (−4.95 to 1.49)
Nursing home		0.66 (−0.56 to 1.87)	0.58 (−0.68 to 1.83)
Other		0.41 (−0.87 to 1.68)	−0.06 (−1.47 to 1.35)
Provider (level 2)			
Unduplicated total number of people supported		−0.0004 (−0.0008 to 0.0001)	−0.0004 (−0.00008 to 0.00007)
Geographic region (ref: urban only)			
Rural only		−1.05 (−2.30 to 0.20)	−0.64 (−1.82 to 0.55)
Both urban and rural		−0.34 (−1.73 to 1.05)	−0.35 (−1.64 to 0.94)
Services provided			
Behavior support services (ref: no)		−0.26 (−1.56 to 1.03)	−0.39 (−1.63 to 0.85)
Therapies (ref: no)		1.06 (−0.26 to 2.39)	1.49 (0.26 to 2.72)^*^
Staffed residential supports (ref: no)		−0.58 (−1.80 to 0.63)	−0.57 (−1.72 to 0.57)
Host home, family foster care, or companion home (ref: no)		−0.25 (−1.52 to 1.03)	−0.57 (−1.76 to 0.61)
In-home supports (own home or family home) (ref: no)		0.62 (−0.47 to 1.71)	0.31 (−0.70 to 1.32)
Community-based employment (ref: no)		0.42 (−0.89 to 1.72)	−0.03 (−1.27 to 1.20)
Community-based day activities (ref: no)		−0.86 (−2.38 to 0.66)	0.21 (−1.27 to 1.70)
In-home day activities (ref: no)		−0.62 (−1.74 to 0.50)	−0.72 (−1.77 to 0.33)
Facility-based work/day activities (ref: no)		−0.25 (−1.41 to 0.91)	0.15 (−0.98 to 1.29)
Respite care (ref: no)		−0.05 (−1.15 to 1.04)	−0.44 (−1.46 to 0.58)
Recreational activities (ref: no)		−0.17 (−1.38 to 1.04)	−0.59 (−1.73 to 0.56)
Transportation activities (ref: no)		0.43 (−0.78 to 1.64)	0.28 (−0.83 to 1.39)
Independent support coordination (ref: no)		0.57 (−0.77 to 1.90)	−0.01 (−1.28 to 1.25)
Basic Assurances®: Total present			0.07 (0.04–0.11)^***^
**Random effects**			
Variance (intercept)	9.83 (7.85–12.30)	9.04 (7.04–11.60)	5.61 (4.12–7.64)
Variance (residual)	18.29 (17.32–19.31)	16.94 (15.91–18.05)	17.92 (16.75–19.18)
χ^2^ (1)	823.42^***^	511.00^***^	271.30^***^
ICC	0.35 (0.31–0.39)	0.35 (0.31–0.39)	0.24 (0.20–0.28)
Cohen's *f*^2^			0.05
***N*** **(people with IDD)**	2,838	2,146	1,848

* *p < 0.05*.

** *p < 0.01*.

*** *p < 0.001*.

Model 2 incorporated the individual-level and provider-level demographic characteristics (Table 3). After adjusting for demographic covariates, the variation in intercepts between providers (ICC) was 34.7%. A number of demographic covariates were significant. Controlling for all other demographic characteristics, women with IDD had fewer personal outcomes present (10.41) than men with IDD (11.07). Controlling for all other variables, people with IDD with assisted decision-making had fewer personal outcomes present (10.36) than people with IDD with independent decision-making (11.07). Controlling for all other variables, Indigenous (9.95), Asian (8.17), and multiracial (8.63) people with IDD had fewer outcomes present than White people with IDD (11.07). Controlling for all other variables, people with IDD with comprehensive behavior support needs (10.08) and *both* complex medical support needs and comprehensive behavior support needs (10.25) had fewer personal outcomes present than people with IDD without any complex support needs (11.07). Controlling for all other variables, compared to people with IDD who lived in provider owned- or operated-homes (11.07), people with IDD who lived in their own home (12.97), family homes (12.57), and host homes or family foster care (13.58) had more personal outcomes present.

**Figure 1:**
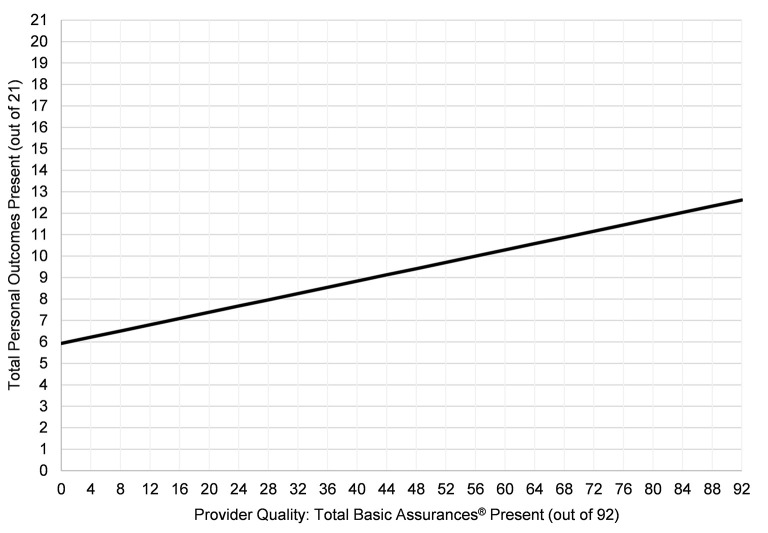
The relationship between provider quality and people with IDD's personal outcomes. Model controls for individual and provider demographics.

Model 3 incorporated provider quality metrics (total Basic Assurances® Table 3). After adjusting for provider quality in Model 3, the variation in intercepts between providers (ICC) reduced to 23.8%, suggesting provider quality partly explains the variation in personal outcomes of people with IDD. The model indicated the more Basic Assurances® indicators their providers had present, the more personal outcomes people with IDD had present—the better the quality of their provider, the better people with IDD's quality of life. For every one Basic Assurance indicator present (out of 92), people with IDD's quality of life increased by 0.07, regardless of their or their providers' demographics (Figure 1). For example, controlling for all individual and provider demographics, a person with IDD served by a provider with a score of 35 on the Basic Assurances® is expected to have 8.5 personal outcomes present (out of 21; 40.3%). Whereas, a person with IDD served by a prover with a score of 70 on the Basic Assurances is expected to have 11.0 personal outcomes present (52.4%).

In addition to provider quality, several demographic covariates were also significant in Model 3. Controlling for all other variables, including provider quality, women with IDD had fewer personal outcomes present (5.32) than men with IDD (5.93). Controlling for all other variables, people with IDD with assisted decision-making had fewer personal outcomes present (5.03) than people with IDD with independent decision-making (5.93). Controlling for all other variables, multiracial people with IDD had fewer outcomes present (3.56) than White people with IDD (5.93). Controlling for all other variables, compared to people without complex support needs (5.93), people with IDD with comprehensive behavior support needs (4.92), and people with both complex medical *and* comprehensive behavior support needs (5.10) had fewer personal outcomes present. Controlling for all other variables, compared to people with IDD who lived in provider owned- or operated-homes (5.93), people with IDD who lived in their own home (7.53), family homes (7.37), and host homes or family foster care (8.41) had more personal outcomes present. Controlling for all other variables, people with IDD who received services from providers that offered therapy services had more outcomes present (7.42) than people with IDD who received services from providers that did not offer therapy services (5.93).

## Discussion

Reinders and Schalock (22) recognize, “quality of life… equals the actualization of discovered potentialities” (p. 293). People with IDD's quality of life is significantly impacted by micro, meso, and macro factors; individual, organizational, and systemic factors simultaneously impact people with IDD's experiences and lives (23). As such, it is important to not only draw attention to people with IDD's personal outcomes, but also the organizational supports they receive to promote those outcomes (3). For these reasons, the aim of this study was to examine the relationship between human service provider quality and people with IDD's personal quality of life outcomes. To do so, we conducted a multilevel linear regression with data from 2,900 people with IDD supported by 331 human service providers. Our findings not only mirror past research which indicates that organizational factors—in additional to individual factors—impact people with IDD's quality of life (10, 22), but also suggest that provider quality *in particular* plays a significant role in people with IDD's personal outcomes.

People with IDD's personal outcomes, regardless of their support needs or other demographics, are significantly impacted by the human service providers they receive services from, and the quality of those providers. As such, provider quality improvement initiatives can significantly improve people with IDD's quality of life. While quality services are multidimensional, people with IDD will not have quality outcomes without a number of foundational elements, including safety, health, and protection from abuse, neglect, and exploitation. Attending to health and safety is particularly important as people with IDD not only face disparities in health, but are also significantly more likely to experience abuse, neglect, mistreatment, and exploitation (24, 25).

Yet, while health and safety are important, they alone do not represent quality services or equal quality of life. Quality services must aim higher than compliance and regulations related to health and safety; instead, quality service provision for people with IDD moves beyond custodial models of care, toward one of a culture that is person-centered, balances duty to care with dignity of risk, promotes informed choice, and honors people with IDD's rights. Provider quality hinges on its commitment to services and supports being responsive to the person—person-centered services and supports. According to self-advocates, “making choices and decisions… is fundamental to having control over our own lives and important for securing all other rights: if we are not allowed to make our own decisions, how can we have a voice in anything else that is important to us?” [(26), p. 65]. Therefore, to ensure services are truly person-centered, providers must have high expectations for all people and ensure people with IDD not only have choices, but also that those choices are informed choices. Informed choice requires people have a variety of life experiences and array of options to choose from.

People with IDD in our study also had better outcomes when their provider offered therapy services (e.g., psychology, occupational therapy, physical therapy, speech language pathology, etc.) as part of the service menu. This finding requires further research, especially as our data did not have information if people with IDD were receiving therapy services or which therapy services they were receiving; we believe it would be especially fruitful to explore if this relationship may be related to trauma-informed care practices. Trauma-informed care not only recognizes a significant number of people with IDD face and experience trauma, but also works to create a “culture that emphasizes safety, trustworthiness, choice, collaboration, and empowerment among service providers and service recipients” [(27), p. 37].

In addition, quality services cannot be provided without adequate and efficient business acumen and processes of human services providers; financial stability of providers is paramount as instability is one of the leading reasons for provider collapse (28). Furthermore, a lack of a consistent and well-trained workforce is a threat to organizational quality, quality improvement initiatives, and, ultimately, the personal outcomes of people with IDD (29).

### Demographic Characteristics and Personal Outcome Disparities

In addition to provider quality, there were a number of individual factors that impacted people with IDD's personal outcomes, which suggests a need for targeted supports. For example, people with assisted decision-making had fewer personal outcomes present than those with independent decision-making. Moreover, women with IDD had fewer outcomes present than men with IDD; this finding mirrors past research which has found women with disabilities, including IDD, experience disparities in quality of life compared to men with disabilities (8) due to the interaction between ableism and sexism. In our study, multiracial people with IDD also had fewer outcomes present than White people with IDD. In fact, controlling for all other variables, multiracial people with IDD only had 16.9% of personal outcomes present on average. Targeted supports are needed for multiracial people with IDD to counter the systemic inequities they face (30).

People with complex support needs—those with comprehensive behavior support needs, and those with complex medical support needs *and* comprehensive behavior support needs—also had disparities in personal outcomes compared to people without these needs. Past research has suggested that the disparities people with higher support needs face are in large part due to a lack of individualized person-centered organizational supports (31). Problematically, a lack of adequate supports and community infrastructure for people with higher support needs often results in re/institutionalization (32).

In addition, there were a number of differences in people with IDD's quality of life based on where they lived. Regardless of support needs, people with IDD had significantly better outcomes when they lived in their own homes, family homes, and host homes/family foster care than in provider owned- or operated-homes. These findings mirror past research about the advantages of these settings, even compared to other community-based settings (7, 33). In fact, in our study, these settings produced better outcomes even when the quality of the providers was controlled. Those settings people with IDD prefer—individualized settings, like their own homes or family homes, rather than congregate settings, such as group homes and institutions—are also the ones that produce the best outcomes (33). As such, providers should make efforts to ensure people with IDD are able to live in individualized settings, should people with IDD wish to do so.

## Limitations

When interpreting the findings of this study, a number of limitations should be noted. This was a secondary data analysis; as such, we did not have the ability to ask participants follow-up questions or add additional variables. There was a large amount of missing data among the variables, which represents a limitation. There may be other individual or organizational factors which were not explored which may impact people with IDD's quality of life. In addition, while it was outside of the scope of this study, there may also be state or regional factors that impacted people's personal outcomes (9). During the COVID-19 pandemic, the use of virtual data collection was more prevalent; the impact of which is unknown and thus represents a limitation of this study. We did not explore interactions in this study. Finally, it should be noted that this is a cross-sectional, correlational study; as such, no causal relationships have been demonstrated.

## Conclusion

People with IDD face a number of disparities in quality of life compared to other populations, largely due to systemic inequities and social determinants of health (25). In this study we found people with IDD who were served by higher quality providers had significantly more personal outcomes present, regardless of their demographics or complex support needs. While quality improvement initiatives may require a significant investment of both time and financial resources from providers (5), our findings suggest the efforts translate to improved personal outcomes among people with IDD. The quality of life of people with IDD demands quality person-centered services and supports. The ultimate goal of service providers should be improvement of quality of life among those they support.

## Data Availability Statement

The original contributions presented in the study are included in the article/supplementary materials, further inquiries can be directed to the corresponding author/s.

## Author Contributions

The author confirms being the sole contributor of this work and has approved it for publication.

## Conflict of Interest

The author declares that the research was conducted in the absence of any commercial or financial relationships that could be construed as a potential conflict of interest.

## Publisher's Note

All claims expressed in this article are solely those of the authors and do not necessarily represent those of their affiliated organizations, or those of the publisher, the editors and the reviewers. Any product that may be evaluated in this article, or claim that may be made by its manufacturer, is not guaranteed or endorsed by the publisher.
